# A Case of Mucosa-Associated Lymphoid Tissue Lymphoma of the Bladder: An Extremely Rare Presentation

**DOI:** 10.7759/cureus.16767

**Published:** 2021-07-30

**Authors:** Shobha Mandal, Chandrakala Dadeboyina, Srijana Baniya Sharma, Suryakala Dadeboyina, Joyson Poulose

**Affiliations:** 1 Internal Medicine, Guthrie Robert Packer Hospital, Sayre, USA; 2 Urgent Care Center, Dr. Paul’s Clinic, State College, USA; 3 Medicine, Rajiv Gandhi Institute of Medical Sciences, Kadapa, IND; 4 Hematology and Oncology, Guthrie Robert Packer Hospital, Sayre, USA

**Keywords:** mucosa associated lymphoid tissue, malt, radiotherapy, urinary bladder, urinary frequency

## Abstract

Extranodal mucosa-associated lymphoid tissue (MALT) is a type of non-Hodgkin’s lymphoma (NHL). It commonly involves the GI tract, but the involvement of the urinary bladder is very rare. It comprises less than 1% of bladder tumors and 0.2% of extranodal lymphomas. Fewer than a hundred cases are reported so far and limited literature is available on the management. Here we are presenting a gentleman with MALT lymphoma of the urinary bladder who presented with urinary frequency, an urgency which was initially managed as benign prosthetic hyperplasia. Initially, the treatment helped him with improvement in his symptoms. After a recurrence of his symptoms 11 years later, he underwent cystoscopy with biopsy that revealed MALT lymphoma of the bladder and underwent radiotherapy.

## Introduction

The malignancy of the urinary bladder is mainly epithelial in origin. In nearly 10-20% of cases, advanced lymphoma of another site metastasizes to the bladder [1]. However, primary lymphoma involving the bladder is very rare. Primary lymphoma of the bladder comprises less than 1% of bladder tumors and 0.2% of extranodal lymphomas [1,2]. Extranodal marginal zone lymphoma of mucosa-associated lymphoid tissue (MALT) accounts for 7-8% of all B-cell lymphoma. In nearly 35% of cases, it involves the stomach but can also affect eyes, ocular tissue, skin, lungs, breast, salivary gland, thyroid, and bladder [3]. The prevalence of secondary lymphoma of the urinary bladder is prominently seen in females compared to males [4]. Here we are presenting a male diagnosed with primary extranodal MALT lymphoma of the bladder.

## Case presentation

**Figure 1:**
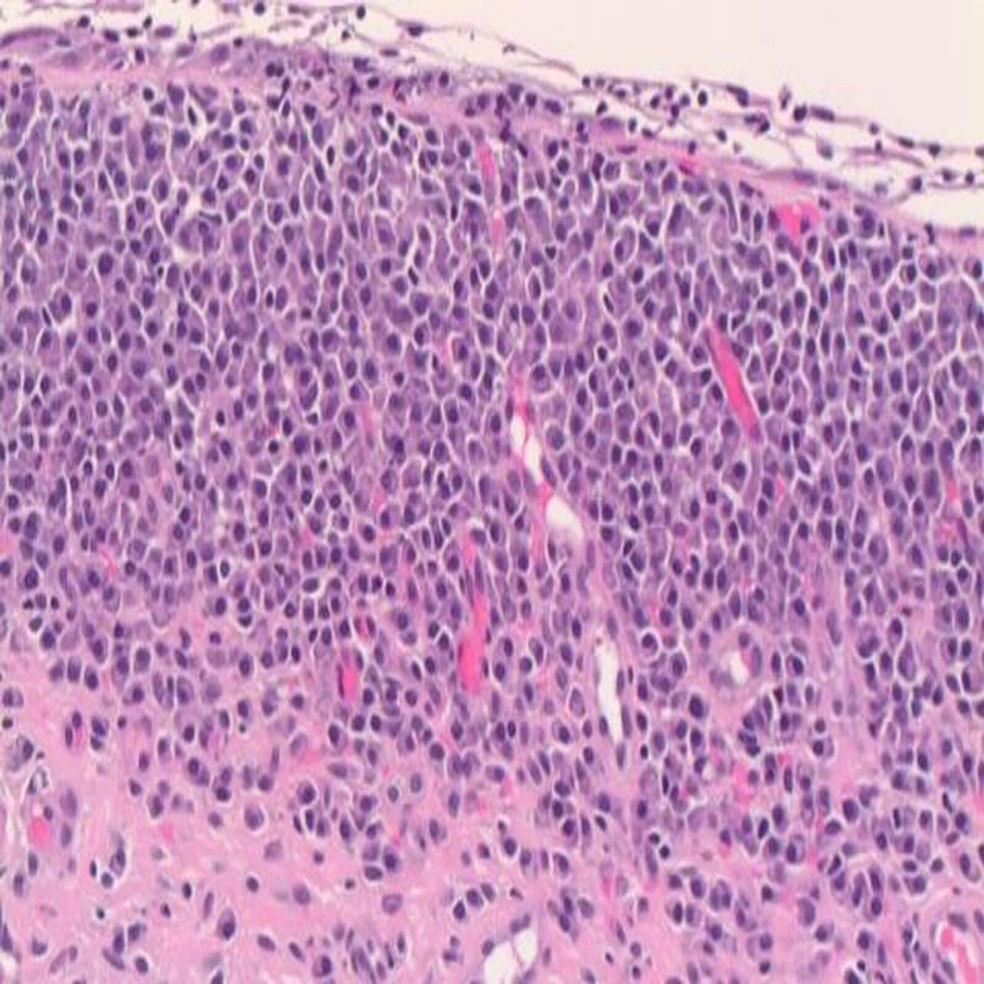
Histopathology showing finding suggestive of MALT lymphoma with extensive plasmacytic differentiation. Denuded urothelium with submucosal mature plasma cell infiltration (H&E X200). MALT: Mucosa-associated lymphoid tissue.

**Figure 2:**
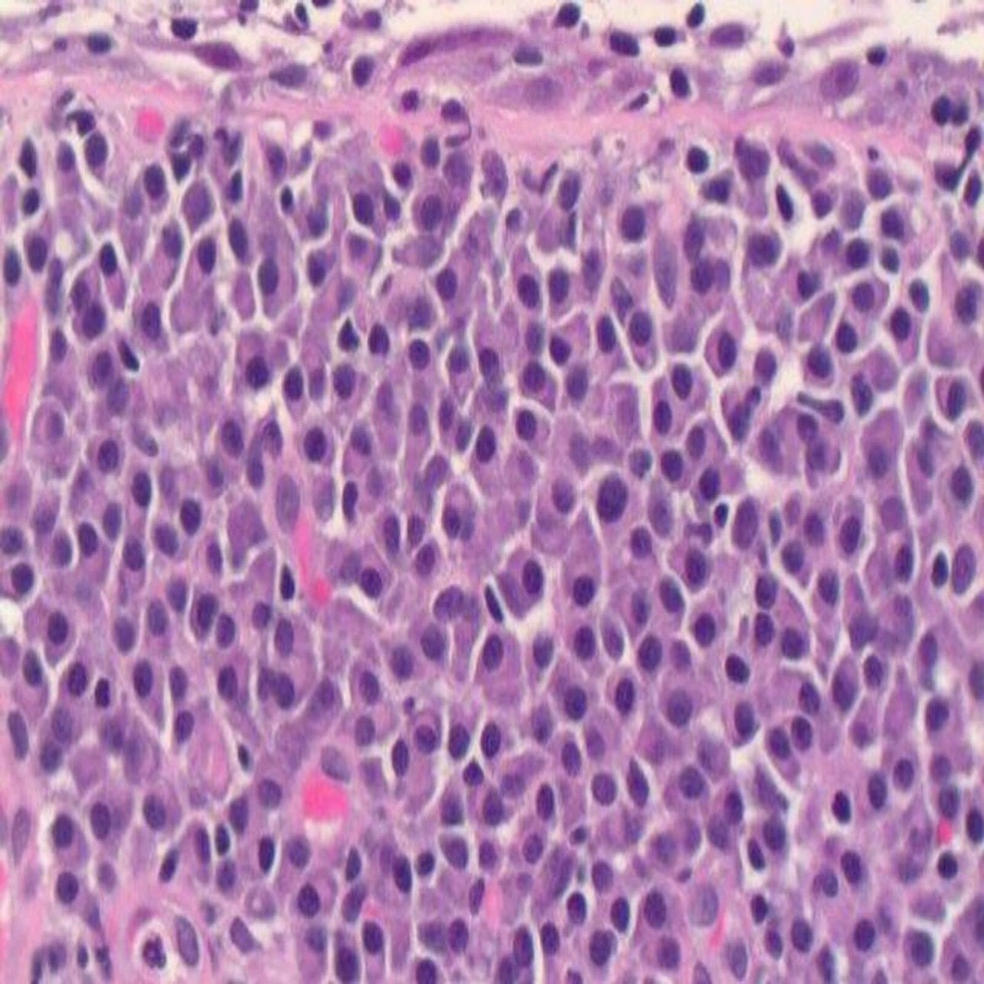
Histopathology showing findings suggestive of MALT lymphoma with extensive plasmacytic differentiation. Denuded urothelium with submucosal mature plasma cell infiltration (H&E X400). MALT: Mucosa-associated lymphoid tissue.

A 78-year-old gentleman with a past medical history of benign prostatic hypertrophy, type 2 diabetes mellitus, and nicotine dependence started having urinary frequency 10 years back. He was treated with tamsulosin and finasteride but had minimal improvement in his symptoms. Later he was treated with solifenacin that improved his symptoms. He was continued on solifenacin and remained symptoms-free until two years ago when he came to see urology with a complaint of reappearance of urinary frequency and urgency. He was continued on medical treatment but was not helpful. He came to see urology one month later with urinary retention which was initially thought to be due to solifenacin and it was stopped. He underwent an ultrasound of the pelvis which showed a normal bladder wall without thickening or trabeculation. Post-void residual volume was 15 ml. He underwent cystoscopy which showed thickening of the lateral wall of prostate and posterior bladder wall erythema. Bladder mass was removed and biopsy was taken from the erythema area which showed findings suggestive of extranodal marginal zone lymphoma of MALT lymphoma with extensive plasmacytic differentiation (Figures 1-2). 

Positron emission tomography-computed tomography (PET/CT) showed no gross evidence of locoregional or distant metastasis. Bone marrow aspirate and biopsy of left iliac bone showed no morphological or immunophenotypic evidence of lymphoma in the bone marrow. A diagnosis of primary MALT lymphoma of the bladder was made based on these findings. A wide array of treatment options, ranging from conservative management, surgery, radiation therapy, and chemotherapy with immunomodulators such as rituximab were presented to the patient. The patient opted for radiation therapy and received one cycle of radiation therapy (2400 cGy in 12 fractions). Patient symptoms gradually improved and a follow-up cystoscopy six months later showed prior resection site with no gross evidence of tumor. The patient was monitored for symptoms.

## Discussion

Malignant lymphoma can either be nodal or extranodal. Extranodal malignant lymphoma most commonly involves the GI tract. Other common sites of involvement are the thyroid and salivary glands. Primary malignant lymphoma involving the bladder is a rare entity that constitutes less than 0.2% of non-Hodgkin’s lymphoma (NHL) and less than 1% of bladder tumors [1]. It is unusual with only fewer than 100 cases in literature so far. Primary bladder lymphoma was first discussed in 1885 by Eve and Chaffey [4,5]. The two most common types of lymphoma are MALT lymphoma and diffuse large B-cell lymphoma. The first reported case of extranodal MALT lymphoma of the bladder was in 1990 by Kempton et al. [5]. Chronic irritation leading to antigenic stimulation of B cells and their proliferation is postulated hypothesis. This mechanism is like MALT lymphoma of GI tissue caused by Helicobacter Pylori, likewise, chronic Hashimoto's thyroiditis leading to a lymphoma of the thyroid gland and Sjogren’s syndrome leading to salivary gland lymphoma [1]. It has a female predominance with a 3:1 ratio. Sixty years and older are affected the most. Nearly 74% of individuals present with hematuria. Other presenting symptoms include dysuria, urinary frequency, and nocturia. 

Given the rarity of this tumor, a limited literature review is present and there is no standard protocol for the management of the tumor. Treatment approaches are variable, and patients were treated with approaches including surgical excision of the tumor, chemotherapy, radiation, antibiotics, or combined modality in different cases with complete remission [6]. A clear-cut consensus is not available about a standard treatment plan for such patients and the efficacy of one treatment over the other hasn't been determined yet. In a few case reports, patients were treated with antibiotics aiming for H. pylori leading to complete remission. Radiation to malignant lymphomas involving the extranodal sites has shown an excellent response [7,8]. Patients of the reproductive age group who are at risk of radiation therapy-induced infertility can be considered for treatment with chemotherapy and immunotherapy. It is believed that patients with systematic spread or recurrence of tumors will benefit from chemotherapy [9,10]. A CD20-specific recombinant monoclonal antibody, rituximab, has shown promising effects with no evidence of recurrence as well [11]. Timely diagnosis and treatment of MALT lymphoma of the bladder have a favorable prognosis. In a few of the cases, the tumor has also transformed into malignant diffuse large B-cell lymphoma [11,12].

## Conclusions

Primary lymphoma involving the bladder is a very rare presentation. MALT lymphoma involving the bladder has a favorable prognosis if treated with chemotherapy, radiation, or surgery timely. Limited literature is available on the presentation and management hence more research is needed to know more about this rare malignancy.
